# Sartans and Bisartans: Expanding Therapeutic Horizons Through Broad Receptor Interactions and Repurposing Potential

**DOI:** 10.3390/ijms27198883

**Published:** 2026-10-06

**Authors:** Errikos Petsas, Vasso Apostolopoulos, Despoina P. Kiouri, Konstantinos Kelaidonis, Harry Ridgway, Graham J. Moore, Thomas Mavromoustakos, Laura Kate Gadanec, John M. Matsoukas, Christos T. Chasapis

**Affiliations:** 1 Laboratory of Organic Chemistry, Department of Chemistry, National and Kapodistrian University of Athens, 15771 Athens, Greece; errpets@chem.uoa.gr (E.P.); despoina.kiouri.99@gmail.com (D.P.K.); tmavrom@chem.uoa.gr (T.M.); 2 School of Health and Biomedical Sciences, RMIT University, Melbourne, VIC 3000, Australia; vasso.apostolopoulos@rmit.edu.au; 3 NewDrug PC, Patras Science Park, 26504 Patras, Greece; costas278264@hotmail.com (K.K.); imats1953@gmail.com (J.M.M.); 4 THERAmolecular, LLC., Rodeo, NM 88056, USA; ridgway@vtc.net; 5 Institute for Sustainable Industries and Liveable Cities, Victoria University, Melbourne, VIC 8001, Australia; 6 Pepmetics Inc., 772 Murphy Place, Victoria, BC V8Y 3H4, Canada; mooregj@shaw.ca; 7 Institute for Health and Sport, Victoria University, Melbourne, VIC 3030, Australia; laura.gadanec@live.vu.edu.au; 8 Department of Chemistry, University of Patras, 26504 Patras, Greece; 9 Department of Physiology and Pharmacology, Cumming School of Medicine, University of Calgary, Calgary, AB T2N 1N4, Canada

**Keywords:** angiotensin receptor blockers, bisartans, G-protein-coupled receptors, polypharmacology, sartans

## Abstract

Angiotensin receptor blockers (ARBs), also known as sartans, are a family of highly selective angiotensin II type 1 receptor antagonists commonly prescribed to individuals with hypertension, heart failure, and chronic kidney disease. Additionally, the discovery and facile synthesis of bisartans, a novel class of ARB antihypertensives, have shown therapeutic promise for cardiovascular diseases. Recent computational and network pharmacology studies have shown a broader receptor interactome, encompassing alpha-adrenergic receptors, and delta- and mu-opioid receptors, highlighting the polypharmacology of ARBs. Herein, we combine molecular docking, molecular dynamics, and protein–protein interaction analyses to characterize the binding profiles of sartans and bisartans, as well as conformational impacts across multiple G-protein-coupled receptors. Emphasis is placed on their potential to modulate receptor desensitization states and influence pathways involved in neuropsychiatric disorders, addiction, viral infections, and cardiovascular diseases. Limitations of current computational approaches are discussed alongside the need for experimental proteomic validation. Collectively, this evidence positions sartans and bisartans as structurally privileged scaffolds for multi-target drug repurposing and precision medicine applications.

## 1. Introduction

Angiotensin receptor blockers (ARBs), commonly referred to as sartans, comprise a cornerstone pharmacological class and are a first-line treatment for hypertension and a wide spectrum of cardiovascular and renal diseases [1,2,3]. The primary mechanism of action of ARBs involves selective competitive antagonism at the angiotensin II (AngII) type 1 receptor (AT_1_R), a G-protein-coupled receptor (GPCR) of the renin–angiotensin–aldosterone system (RAAS), exhibiting compound-dependent surmountable or insurmountable functional behavior. Whereas first-generation agents such as losartan display classically surmountable antagonism, several other ARBs (e.g., candesartan, telmisartan, and olmesartan) demonstrate insurmountable antagonism, primarily driven by high receptor affinity and slow dissociation rates from the orthosteric AT_1_R pocket, thus inhibiting the vasoconstrictive [4], pro-inflammatory [5], and aldosterone-stimulating actions [4] of AngII [6]. This blockade leads to systemic vasodilation, decreased fluid retention, and, ultimately, reduced arterial pressure and cardiac workload [6]. Compared to angiotensin-converting enzyme inhibitors [7], ARBs demonstrate a more favorable safety profile [2]. Notably, they do not interfere with bradykinin degradation [8], which minimizes adverse effects, such as dry cough and angioedema [9]. Beyond classical antagonism, many ARBs function as inverse agonists at AT_1_R, stabilizing its inactive conformation [10,11]. This activity has been linked to receptor internalization and desensitization mechanisms that dampen receptor signaling and contribute to long-term therapeutic efficacy. These effects may be modulated by receptor cooperativity, a phenomenon wherein ligand binding to one subunit of a dimeric or oligomeric receptor complex influences the binding affinity and activity at adjacent sites [12]. In this context, inverse agonism and negative homotropic cooperativity may reduce receptor responsiveness and signaling output.

Beyond functioning as a classic endocrine axis regulating systemic hemodynamics, the RAAS operates via widespread local and tissue-specific networks with vital autocrine and paracrine actions. Chronic dysregulation of the canonical angiotensin-converting enzyme (ACE)/AngII/AT_1_R axis relative to counter-regulatory pathways, notably the ACE2/angiotensin (1–7)/Mas receptor axis, drives pro-inflammatory cascades, oxidative stress, endothelial dysfunction, and progressive tissue fibrosis [1,2,3]. Consequently, aberrant tissue-specific RAAS signaling serves as a critical pathological driver across a wide spectrum of disorders beyond uncomplicated hypertension. These include adverse cardiovascular remodeling and cardiac hypertrophy, progressive chronic kidney disease and diabetic nephropathy, acute lung injury and virus-induced pulmonary inflammation, retinal microvascular degeneration in diabetic retinopathy, and neuro-inflammatory processes orchestrated by the local brain RAAS. Hyperactivity of the systemic and tissue RAAS has also been deeply implicated in peripheral insulin resistance, metabolic syndrome, and non-alcoholic fatty liver diseases. While sartans do not represent standard clinical treatments for all these pathologies, understanding how systemic and tissue RAAS networks fuel chronic inflammation and fibrotic remodeling provides a sound biological rationale for exploring ARBs and bisartans as versatile candidates for pharmacological repurposing [5].

Recent advances in computational pharmacology, particularly molecular docking, molecular dynamics (MD) simulations, and systems-level network modeling, have provided new insights into the dynamic behavior and structural versatility of ARB-receptor interactions [13,14]. In silico studies suggest that sartans, and their chemically enhanced analogs (bisartans), exhibit noncanonical binding at AT_1_R and may also engage with a broader range of GPCRs, including α-adrenergic receptors (αARs), and mu- (μOR) and delta-opioid receptors (δOR) [15]. These findings highlight a potential polypharmacological profile, with implications for receptor crosstalk and downstream signaling diversity [15].

Importantly, this expanded receptor interactome offers opportunities for drug repurposing in complex diseases beyond cardiovascular indications [16,17,18], including neuro-inflammatory disorders [19,20], substance use disorders, and viral infections, such as coronavirus disease 2019 (COVID-19) [20]. These phenomena are increasingly studied through multiomics and structural proteomics approaches, which support the mechanistic investigation of drug–proteoform interactions and facilitate the mapping of pharmacologically relevant protein networks.

This review compiles existing knowledge on the broad pharmacological landscape of ARBs and bisartans, concentrating on newly identified receptor targets and signaling pathways identified through computational modeling, receptor profiling, and systems pharmacology. Specific focus is placed on the translational potential of these findings, highlighting logical paths for therapeutic repurposing based on emerging insights into receptor–ligand dynamics and proteoform-specific interactions.

## 2. Receptor Binding of Sartans and Bisartans

### 2.1. Broad Receptor Binding Profiles of Sartans and Bisartans

**Figure 1:**
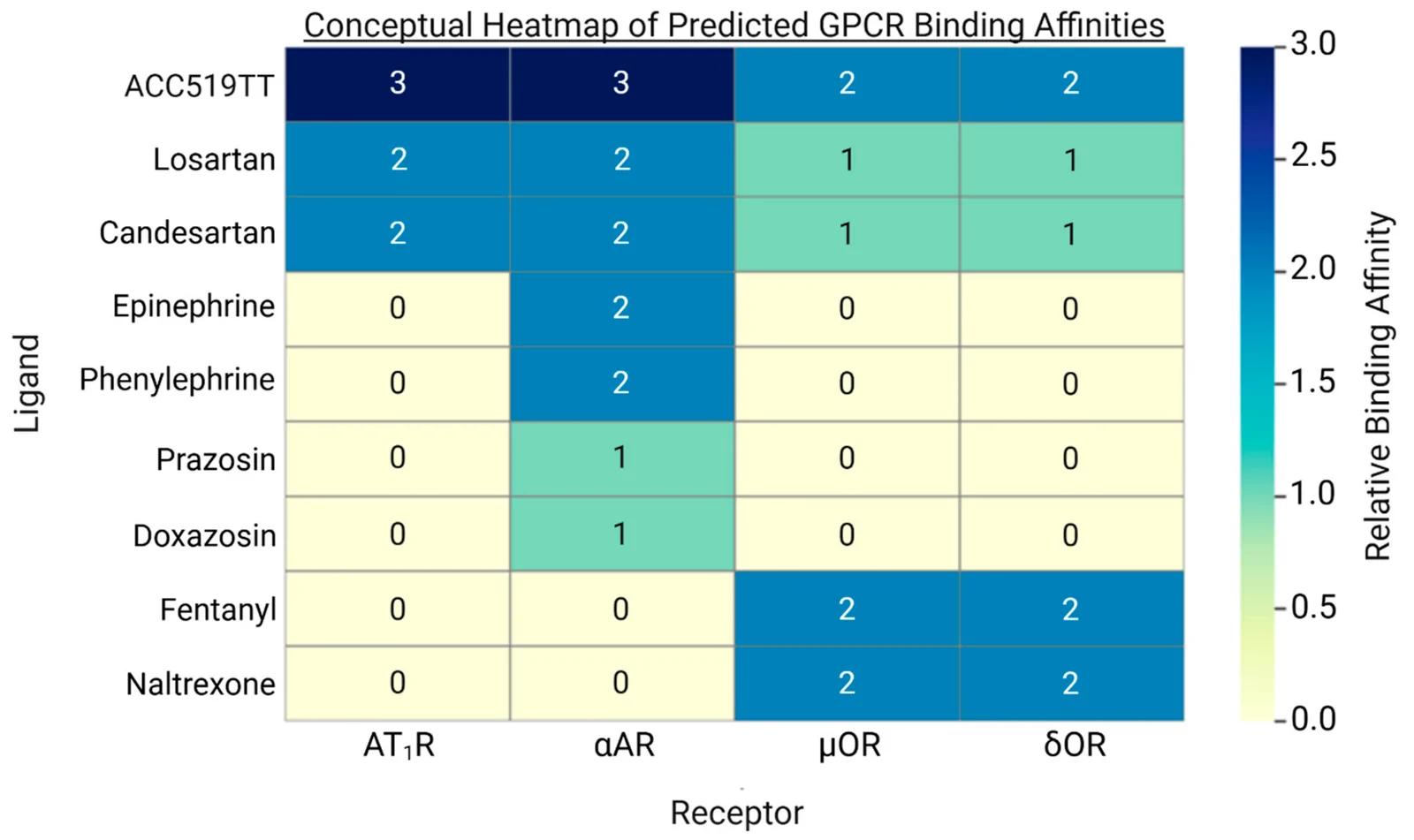
Semi-quantitative heatmap of predicted ligand binding affinities for selected GPCRs. Relative binding strength was inferred from molecular docking and MD data, comparing ARBs with canonical ligands. ACC519TT exhibited strong predicted binding to AT_1_R and αARs, and moderate binding to μOR and δOR. Affinity values were categorized as: high = 3; moderate = 2; low = 1 and non-binding scored = 0.

Computational studies, including molecular docking and MD simulations, revealed that ARBs, comprising both traditional sartans and their optimized derivatives, bisartans, exhibit unexpectedly broad receptor binding profiles [15]. In addition to their canonical target, the AT_1_R, these compounds demonstrate notable predicted affinities for αARs, μOR, and δOR (Figure 1) [15].

Among the compounds assessed, the bisartan, benzimidazole bis-N,N′-biphenyl tetrazole (ACC519TT), consistently exhibited higher docking scores and computed binding energies for AT_1_R compared to conventional sartans [15]. Surprisingly, several ARBs also showed stronger binding predictions for αARs than classical ligands, including both agonists (e.g., epinephrine, phenylephrine) and antagonists (e.g., prazosin, doxazosin) [15]. Docking scores for ARBs at μOR and δOR were moderate to high, in some cases surpassing those of reference ligands (e.g., fentanyl and naltrexone) [15]. However, the absolute binding affinities for opioid receptors were significantly lower than those observed for AT_1_R and αARs, suggesting potential pharmacological relevance only under specific physiological or pharmacokinetic conditions [15].

In the underlying computational studies [15], molecular docking was executed using a targeted, grid-based protocol centered on the defined orthosteric binding pockets near the extracellular domain. Consequently, ligand poses predominantly occupied these predefined regions, aligning with the binding cavities of endogenous agonists rather than identifying novel allosteric sites via unbiased blind docking. Within these pockets, binding was stabilized through a combination of hydrophobic contacts, hydrogen bonding, π-π stacking, and electrostatic forces (e.g., cation–π and salt bridges) [15]. Specifically, in the AT_1_R-ACC519TT complex, stabilizing contacts included cation–π interactions with Arg23 and Arg167, alongside aromatic stacking with Tyr35 and Tyr92 [15].

ACC519TT was chosen for MD simulations to evaluate the stability of the predicted ligand–receptor complexes. Its selection was based on its strong docking scores and structural complexity, making it ideal for studying the dynamic behavior of these interactions over time [15]. Across 150–200 ns trajectories, the ligand remained stably bound within the orthosteric pockets of AT_1_R, μOR, and δOR [15]. Complexes with δOR and AT1R displayed comparable overall backbone root-mean-square deviation (RMSD) values of 3.08 Å and 3.02 Å, respectively, whereas the μOR complex showed an RMSD of 2.47 Å. In the δOR simulation, an accompanying counterclockwise rotation of the transmembrane bundle was observed, which was interpreted as an antagonist-like profile [15].

These findings support the concept that ARBs, particularly bisartans, may exhibit a broader pharmacodynamic repertoire than previously appreciated [15]. Their ability to engage distinct GPCR families and induce receptor-specific structural effects points to a polypharmacological potential with therapeutic implications beyond classical cardiovascular applications. However, backbone RMSD values within nanosecond timescales reflect predicted ligand-induced conformational flexibility rather than definitive functional efficacy (agonism, partial agonism, or inverse agonism). Establishing functional modality strictly requires direct evaluation of conserved GPCR microswitches (e.g., CWxP, DRY, NPxxY motifs), effector coupling (G-protein vs. β-arrestin recruitment), and experimental functional signaling assays [15].

### 2.2. Overlapping Bindings Sites and a Gating Mechanism at AT_1_R

The ligand–receptor interactions at AT_1_R can be described within an extended conformational selection framework encompassing three principal functional states: an active signaling state (R_active_), a basal/inactive ground state (R_inactive_), and a desensitized/internalized state (R_des_) [21,22]. Ligands selectively shift this conformational equilibrium toward distinct signaling or regulatory outcomes.

AngII, the endogenous AT_1_R agonist, preferentially stabilizes the R_active_ conformation, facilitating G-protein coupling, positive cooperativity, and classical vasoconstrictive cascades. In contrast, ARBs act as inverse agonists that suppress basal signaling and favor the R_des_ state, promoting receptor internalization and desensitization, potentially mediated by β-arrestin engagement [23,24,25].

**Figure 2:**
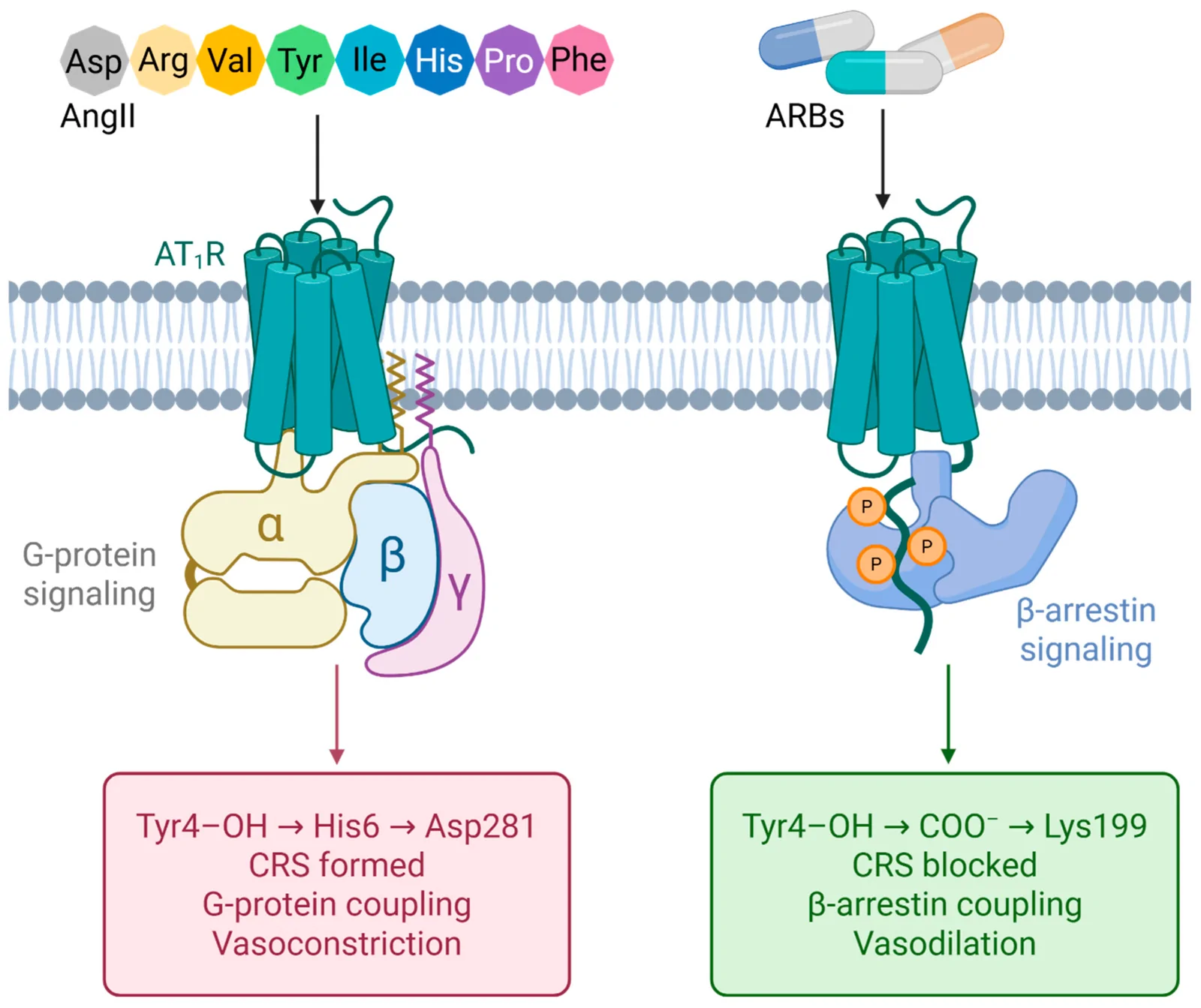
Ligand-specific binding and signaling outcomes at the AT_1_R. AngII binds in the R_active_ conformation [23], enabling CRS formation through interactions between Tyr4, His6, and Phe8, ultimately leading to G-protein coupling and vasoconstriction. In contrast, ARBs compete with and displace AngII across overlapping orthosteric binding regions, engaging ligand-specific residues (such as Lys199) and stabilizing the Rdes conformation to disrupt CRS formation, promote beta-arrestin recruitment, and attenuate AngII-induced vasoconstrictor tone [23]. Despite targeting an overlapping orthosteric pocket, peptide agonists and nonpeptide ARBs stabilize distinct functional states through divergent intramolecular contacts. Created using BioRender. Gadanec, L. (2026) https://BioRender.com/obpwya2 (accessed on 25 September 2026).

A central feature of AngII–AT_1_R interaction is a charge relay system (CRS) [26]. In solution, AngII undergoes internal proton transfer from the hydroxyl of Tyr4 to His6 and ultimately to the C-terminal carboxylate of Phe8 [27], generating a tyrosinate anion necessary for high-affinity receptor binding [26]. Upon docking, this CRS is extended through intermolecular interactions with Asp281 of AT_1_R [28] mimicking the role of Phe8. This creates a molecular switch controlling receptor activation. Two ligand-specific outcomes emerge from this mechanism (Figure 2):

i. Agonist binding (e.g., AngII): enables CRS formation via Tyr4–His6 interaction (facilitated by Asp281), promoting R_active_ signaling.
ii. Inverse agonist binding (e.g., sartans): competes with and displaces AngII through overlapping orthosteric regions, disrupting the CRS by redirecting Tyr4-OH toward the C-terminal group and establishing ligand-specific contacts (such as a salt bridge with Lys199) that selectively stabilize the desensitized receptor conformation (Rdes).

The Phe8 ring further influences receptor activation by stabilizing Arg2, directing Asp281 to activate His6. Conversely, ARBs bypass this gating mechanism and interact directly with Arg167 [29,30]. Bisartans, containing two biphenyl tetrazole groups, may simultaneously contact both Arg167 and Lys199, enhancing binding stability and potentially expanding receptor engagement.

This mechanistic model highlights that AT1R activation and inhibition are determined not only by binding affinity but also by intramolecular CRS that modulate conformational state and signaling behavior.

### 2.3. New Receptors and Repurposing Implications

Sartans and bisartans show potential for repurposing due to their ability to bind noncanonical targets, such as αARs and μORs, beyond their traditional cardiovascular applications. To investigate wider pharmacological possibilities, a systems-level interactome analysis was conducted, including first-neighbor protein–protein interactions of established ARB targets [31].

**Figure 3:**
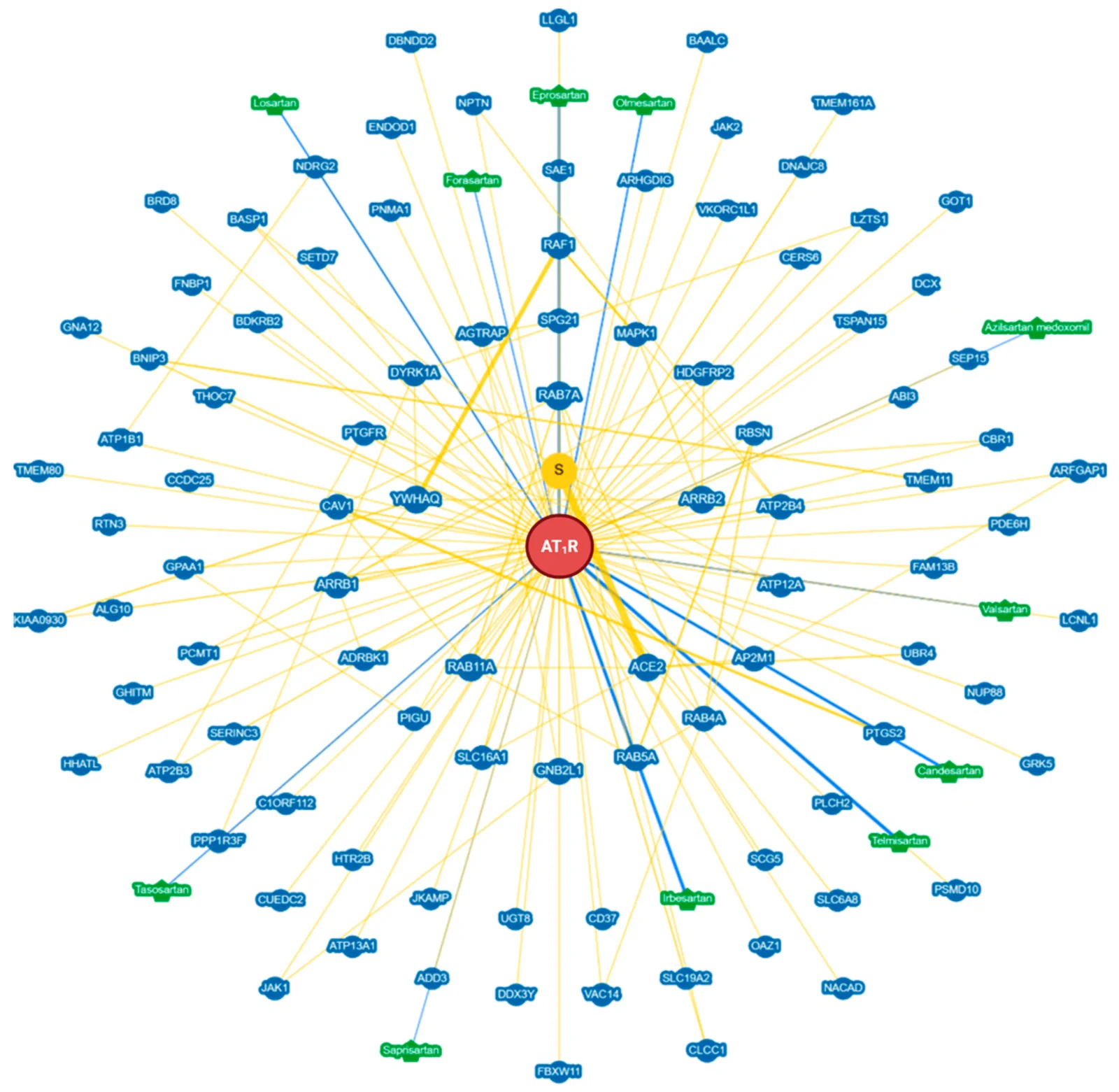
The protein and drug (i.e., sartans) interaction network of AT_1_R, the common receptor of all sartans and bisartans, visualized using BioGrid (version 4.4.248) (https://thebiogrid.org/ accessed: 11 July 2025) [34,35].

Primary targets include: AT_1_R (shared by all ARBs) (Figure 3); Jun proto-oncogene, AP-1 transcription factor subunit (c-JUN) (notably for irbesartan); peroxisome proliferator-activated receptor gamma (PPARγ) (telmisartan); ACE2 (the receptor for severe acute respiratory syndrome coronavirus 2 (SARS-CoV-2) [32,33]). AT_1_R was found to interact with 48 proteins, c-JUN with 186, PPARγ with 120, and ACE2 with nine [31].

Functional enrichment of these networks revealed:

i. Conventional ARB use: Enriched functions include transcription factor activity, domain-specific binding, and acetyltransferase activity [31].
ii. Antiviral/repurposing context (ACE2 + AT_1_R): Enrichment in GPCR binding, exogenous protein interaction, adenylate cyclase modulation, and phosphatase activation—functions associated with host–virus dynamics and immune signaling [31].

Disease-association analysis linked ARB-target networks with various conditions:

i. As antihypertensives: Breast cancer, myelofibrosis, Von Hippel–Lindau syndrome, Burkitt lymphoma [31].
ii. In SARS-CoV-2 context: Breast carcinoma, melanoma, hypertensive crises, ventricular tachycardia [31].

Emerging preclinical studies indicate the potential for ARBs in addiction therapeutics [36,37]. Male Wistar rats co-treated with morphine (10 mg/kg) and valsartan (20 mg/kg) for 7 consecutive days displayed reduced signs of morphine withdrawal (i.e., withdrawal jumps, defecation and diarrhea) when compared to rats only receiving morphine [36]. Additionally, candesartan (1µM/L and 5 µM/L) inhibited AT_1_R expression in morphine-treated BV2 microglial cells (200 µM) and dose-dependently prevented expression of inflammatory markers (i.e., tumor necrosis factor alpha, interleukin-6 and interleukin-18) and cell activation through the PPARγ/AMPK signaling pathway [37]. Taken together, these studies support the ability of sartans to protect against morphine tolerance and dependence, highlighting an important and poorly understood role that the AT_1_R has in opioid addiction.

**Figure 4:**
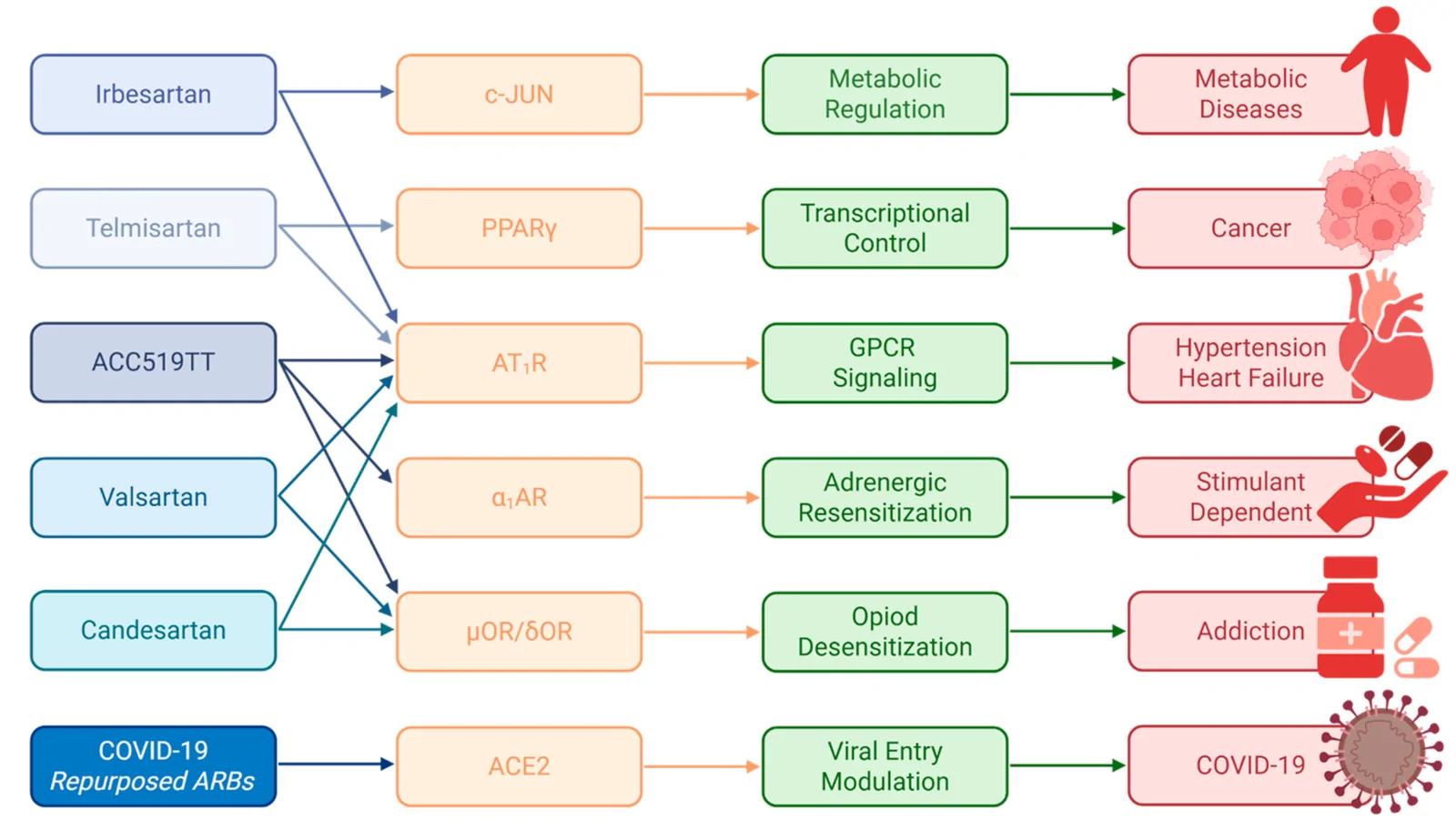
Schematic representation of drug–target–function–disease relationships for sartans and bisartans. This layered flowchart illustrates the repurposing rationale of ARBs and their second-generation derivatives (bisartans), showing how individual compounds (**left**) affect specific molecular targets (**center–left**) and elicit distinct biological functions (**center–right**) that may impact disease pathogenesis and development (**right**). Sartans, such as candesartan, irbesartan, telmisartan and valsartan, not only target the AT_1_R but also demonstrate the ability to directly associate with and indirectly influence off-targets, including ACE2 [38,39], c-JUN [40,41], opioid receptors [36,37] and PPARγ [42,43,44]. Importantly, the bisartan compound, ACC519TT, shows multi-target binding to GPCRs, including AT_1_R, α_1_AR, µ- and δORs [15]. These interactions modulate functional pathways, including GPCR signaling, metabolic and transcriptional regulation, viral entry, and opioid/adrenergic receptor desensitization. The resulting pharmacological activities suggest repurposing opportunities for treating metabolic diseases, cancer, COVID-19, addiction, and stimulant dependence, beyond the classical cardiovascular indications. Created in BioRender. Gadanec, L. (2026) https://BioRender.com/u0a5p4j (accessed on 25 September 2026).

Such modulation is particularly relevant in addiction, where agonist-induced receptor desensitization contributes to tolerance. Since opioid agonists have similar affinity for both R_active_ and R_des_ receptor states, ARBs could selectively engage the R_des_ without triggering euphoric responses, disrupting desensitization cycles non-addictively. These findings highlight bisartans as promising candidates for repurposing in addiction and stimulant-use disorders, grounded in their ability to modulate receptor desensitization dynamics and cross-GPCR signaling pathways (Figure 4).

To delineate computational predictions from experimental validation and summarize compound-specific profiles across these diverse pathways, individual sartans and bisartans are systematically compared in Table 1.

## 3. Future Directions

Pharmacological blockade of the RAAS offers a therapeutic strategy across multiple cardiovascular and renal conditions [1,2,3]. The RAAS is a multifaceted endocrine system that is the main coordinator of blood pressure and fluid homeostasis by regulating vascular resistance and tone, absorption and excretion of electrolytes, and hemodynamic flow and volume [48,49]. AngII predominantly acts via AT_1_R to raise blood pressure through vasoconstriction, sympathetic activation, aldosterone secretion and enhanced sodium retention. Under normal homeostatic balance, this is counteracted by an opposing non-traditional arm whereby AngII is acted upon by ACE2 [50]. This results in cleavage of the Phe amino acid at position 8, which is the critical amino acid of AngII for agonist activity. For example, the beneficial effect of the protective arm of the RAS includes ACE2-dependent cleavage of Phe position 8 of angiotensin A or decarboxylation of angiotensin 1–7 (a product of ACE2 conversion of AngII) to generate the cardioprotective heptapeptide, alamandine [51,52,53].

Emerging computational and systems pharmacology evidence reveals that sartans and bisartans interact not only with their canonical target, the AT_1_R, but also with a broader network of GPCRs, including αARs and μORs [15]. These findings challenge the traditional paradigm of selective ligand–receptor binding and position ARBs as promiscuous, multi-target agents with significant potential for drug repurposing across therapeutic domains [31,54].

The structural characteristics of sartans and bisartans underpin their polypharmacological behavior. Most members of this class feature a positively charged or partially charged imidazole/imidazolium core integrated into a hydrophobic scaffold [55]. Bisartans, bearing formal positive charges, display stronger predicted binding than conventional sartans, likely due to enhanced electrostatic interactions. Additionally, their anionic tetrazole groups, known to mimic carboxylate functionalities, offer versatile interaction profiles, supporting hydrogen bonding, ionic interactions with cationic residues (e.g., Arg, Lys), and π-stacking with conserved aromatic residues in GPCR binding pockets [15].

### 3.1. Therapeutic Implications of Receptor Promiscuity

#### 3.1.1. Cardiovascular Applications

The dual antagonism of AT_1_R and αARs by certain ARBs suggests the potential for synergistic antihypertensive effects. This dual targeting may outperform monotherapy with either ARBs or αAR-blockers alone, especially in patients with autonomic dysregulation. Furthermore, the reciprocal modulation of AT_1_R-αAR signaling suggests a systems-level opportunity to optimize vascular tone via combined pathway suppression.

#### 3.1.2. Addiction and Neuropsychiatric Disorders

Several sartans, including valsartan and candesartan, have been shown to attenuate the development of morphine tolerance in rodent and cellular models, likely by modulating GPCR desensitization pathways. Opioid agonists typically exhibit comparable affinity for both R_active_ and R_des_ receptor states, leading to receptor downregulation and functional tolerance. ARBs, particularly bisartans, may selectively stabilize the desensitized conformation without triggering receptor activation, functioning as non-euphoric modulators. This mechanism supports the repurposing of these compounds as adjuncts in the treatment of opioid or stimulant dependence.

#### 3.1.3. COVID-19 and Viral Infections

Interest in repurposing sartans for COVID-19 is driven by their potential to reduce morbidity and mortality in patients and to modulate ACE2 expression [56,57,58]. Network-based pharmacology analysis reveals that ARBs interact with protein–protein interaction networks comparable in scale and connectivity to approved antivirals, such as Paxlovid. However, contradictory clinical evidence [46,59] and enrichment of ARB-interacting proteins in cancer [60,61] developmental pathways (e.g., pregnancy loss) [62,63] warrant rigorous safety profiling, especially for vulnerable populations.

### 3.2. Methodological Considerations: From CAD to Proteoform Function

One major challenge in translating in silico results into biological relevance stems from the limitations of receptor models used in computer-aided drug design (CAD) [64]. Most CAD frameworks utilize static, uncoupled (“dead”) receptor conformations that lack intracellular signaling components. These models often overestimate ligand affinity, particularly for inverse agonists and desensitization-stabilizing ligands, which may preferentially bind receptor proteoforms associated with inactive or internalized states. Nevertheless, CAD results provide valuable insights, particularly when targeting desensitized or noncanonical receptor states for which experimental structures are unavailable. Ligand-specific conformational stabilization observed in MD simulations (e.g., δOR bundle rotation) reinforces the utility of these approaches for probing conformational plasticity and functional selectivity.

To transcend the constraints of conventional grid-based docking and standard nanosecond-scale MD simulations, future computational exploration of sartan and bisartan polypharmacology must leverage modern artificial intelligence paradigms alongside advanced statistical mechanics. While conventional docking indicates putative binding poses, it inadequately samples receptor plasticity and free energy landscapes. The integration of enhanced sampling methodologies, such as well-tempered metadynamics, replica-exchange molecular dynamics, and umbrella sampling with potential of mean force calculations, will be crucial to compute rigorous binding free energies, elucidate unbinding kinetics (k_off_), and map the free energy barriers governing transitions between R_active_, R_inactive_, and R_des_ states. Furthermore, the advent of deep learning architectures, notably AlphaFold-Multimer, ESMFold, and RosettaFold-All-Atom, offers unprecedented capabilities to generate high-confidence structural ensembles of fully coupled GPCR-transducer complexes (including heterotrimeric G_αβγ_ proteins and β-arrestins). Applying geometric deep learning and generative diffusion models tailored for ligand-directed conformational ensembles could substantially refine multi-target virtual screening across adrenergic and opioid receptor families.

Crucially, in silico hypotheses must be paired with rigorous biophysical and functional validation to establish pharmacological relevance. Experimental workflows should transition from purely descriptive computational metrics to orthogonal biophysical assays, such as surface plasmon resonance or bio-layer interferometry for real-time kinetic determination (k*on*, k*off* and residence time), alongside cryo-electron microscopy to visualize definitive bisartan binding modes within ternary GPCR complexes. Ultimately, ligand-induced receptor modulation requires comprehensive cellular profiling across canonical and noncanonical pathways. This entails second-messenger quantification (homogeneous time-resolved fluorescence for cAMP accumulation and IP-One for G_q_-mediated inositol phosphate turnover) coupled with bioluminescence resonance energy transfer-based biosensors to directly evaluate β-arrestin-1/2 recruitment and receptor internalization dynamics. Such an integrated pipeline will critically distinguish inert off-target binding from functionally meaningful, biased GPCR modulation.

### 3.3. Structural Insights and Proteoform Selectivity

The strong binding affinities observed for bisartans are structurally supported by a multi-valent pharmacophore architecture. The tetrazole group acts as both an ionic and π-stacking partner, while central benzimidazole or imidazolium rings enable cation–π and hydrophobic interactions across multiple GPCRs. This versatility allows bisartans to engage diverse receptor proteoforms, possibly including receptor isoforms, splice variants, or post-translationally modified conformers relevant to specific disease contexts. Such proteoform-level selectivity may underlie the diverse pharmacodynamic profiles observed for these compounds and emphasizes the importance of future work integrating experimental proteomics (e.g., targeted mass spectrometry or structural proteomics) to validate receptor-specific binding and signaling consequences.

### 3.4. From AngII to Sartans and Bisartans: The CRS and the Role of Tyrosine to Trigger Hypertension and Cardiovascular Diseases

Building upon the charge relay framework described in Section 2.2, seminal research demonstrated that ${\mathrm{AT}}_{1}R$ activation critically relies on the formation of the pharmacophoric tyrosinate anion through the aromatic triad (Tyr, His, and Phe) and the C-terminal carboxylate [65,66]. The essential functional role of the Tyr4 hydroxyl in triggering vasoconstrictive and hypertensive signaling cascades was validated by methylation studies: the resulting analog, [Sar1Tyr(Me)4] AngII (sarmesin), exhibited a complete loss of agonist efficacy and acted as a competitive antagonist [67,68]. Subsequent crystallographic investigations corroborated this direct interaction between the tyrosine hydroxyl and phenylalanine carboxylate within AngII [30,69,70,71,72,73], confirming the catalytic role of the Tyr hydroxylate in receptor activation [27,74]. Furthermore, the Arg2 guanidinium side chain chaperones the aromatic ring cluster, stabilizing the charge relay architecture via electrostatic and π–π interactions [75,76]. This active geometry is structurally constrained by Pro7, a sterically hindered residue that induces a vital γ-turn in the peptide backbone essential for bioactivity [75,76].

### 3.5. Polyfunctional Properties of Sartans

Other studies stating the polyfunctional properties of sartans have shown that telmisartan effectively inhibits AT_1_R in glioma cell lines [47]. In general, sartans, like candesartan, manifest their activity by warhead anionic tetrazoles or carboxylates, mimicking AngII tyrosinates. The resulting binding blocks the activity of positive arginines that trigger SARS-CoV-2 infection [77,78,79]. Furthermore, studies have also investigated the relationship of ARBs with autoimmune diseases, such as multiple sclerosis and experimental autoimmune encephalomyelitis. These studies have shown that ARBs exhibit anti-inflammatory, neuroprotective and immunomodulatory properties, making them candidates for repurposing in neuron conditions [17,80,81]. In particular, myelin basic protein antigens, implicated in multiple sclerosis, contain many arginines, basic in nature with a positive charge, and have been extensively studied by our laboratory group to investigate cytokine secretions in patients with multiple sclerosis patients [82,83]. The abundant content of basic arginines in myelin basic protein (a total of 19 arginine residues) makes it and its antigens an attractive therapeutic target to investigate the effects of ARBs and bisartans containing warhead anionic tetrazoles that bind strongly to arginines [84,85].

### 3.6. Bisartans: Second-Generation Nonpeptide Mimetics of AngII as Antihypertensives and Pan-Antiviral Drugs

Second-generation ARB bisartans, in which both imidazole nitrogens are substituted with biphenyl tetrazole moieties, demonstrated favorable computed interaction energies and predicted binding within the catalytic pockets of SARS-CoV-2, influenza, and respiratory syncytial virus targets in in silico models [77]. This property may derive from the dual interaction of both warhead negative tetrazoles with positive arginines, which trigger infections [15,77,86,87]. Elevated predicted docking scores for bisartans across opioid and adrenergic receptor models have been attributed to favorable electrostatic interactions involving the tetrazole moieties [15]. Previous research on the design of losartan analogs has led to the discovery of a new class of ARBs where the imidazole substituents, butyl and hydroxyl methylene groups at positions 2 and 4, respectively, are at reversed positions compared to losartan [88]. These analogs were the basis to further develop bisalkylated derivatives that symmetrically bear two biphenyl tetrazole groups on the two imidazole nitrogens (bisartans), with notable properties relevant to hypertension and COVID-19 therapies [45,89].

### 3.7. Benzimidazole Sartans

The use of benzimidazole as a scaffold instead of imidazole and bis-biphenyl tetrazole alkylation resulted in the development of novel bisartans, which exhibited unique binding affinities due to the two biphenyl tetrazoles and increased aromaticity caused by the benzimidazole fused rings [45]. Interaction of aromatic phenyl groups with arginines, as between ARBs and AT_1_R Arg167, has been previously reported by our laboratory group as a dominant binding factor due to the π-π electron interactions [15,45,87,90]. Arginine is a key amino acid in triggering disease and arginine blockers, like ARBs or bisartans containing warhead anionic tetrazoles, represent candidate chemical entities for further investigation against arginine-rich viral domains and off-target GPCR signaling pathways [15]. Additionally, arginines can interact with tetrazoles in aromatic rings, such as phenylalanine, increasing binding [91,92]. The physicochemical and bioisosteric properties of the tetrazole scaffold continue to receive extensive investigation in medicinal chemistry for multi-target drug design [93,94,95].

### 3.8. Peptide Versus Nonpeptide Ligands (Sarmesin and Sarilesin Veruses ARBs)

**Figure 5:**
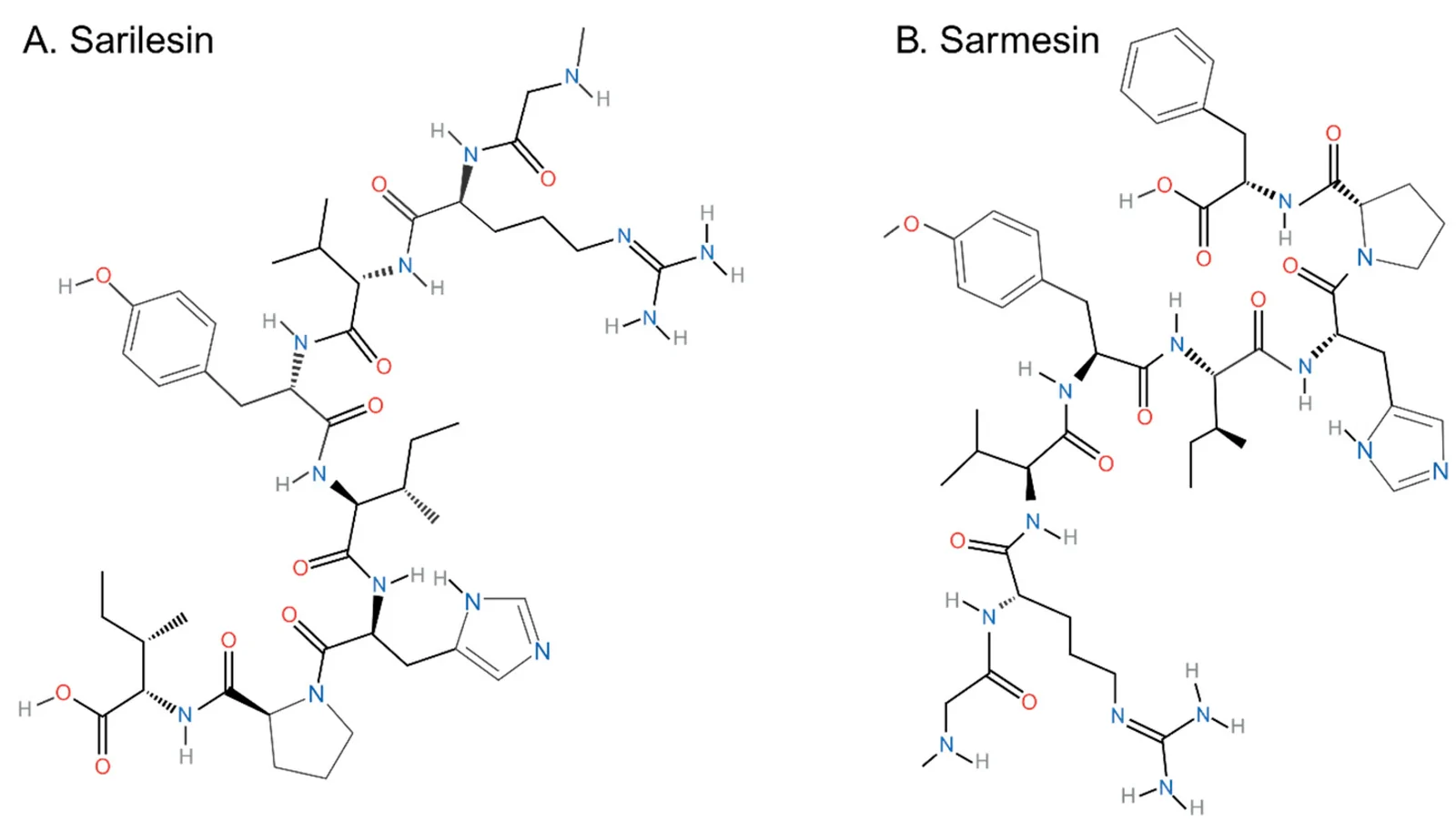
Chemical representation of (**A**) sarilesin and (**B**) sarmesin. Created in BioRender. Gadanec, L. (2026) https://BioRender.com/9h63qrt (accessed on 25 September 2026).

The synthesis of analogs of the octapeptide AngII demonstrated that substitution of the Asp1 residue with Sar1 increased activity, and that replacement of the Phe8 residue of AngII with an aliphatic amino acid, as in [Sar1Ile8]AngII (sarilesin) (Figure 5A), produced a noncompetitive antagonist (later assigned as inverse agonism), whereas methylation of the Tyr4 hydroxyl group led to the competitive antagonist [Sar1Tyr(Me)4]AngII (sarmesin) (Figure 5B). Methylation of the Tyr4-OH of the inverse agonist sarilesin also expunged bioactivity, illustrating that the Tyr hydroxyl proton [and/or tyrosinate anion] is important for both agonist and inverse agonist actions. Conformational investigations also pointed to the presence of a charge relay interaction in AngII, in which the Tyr4 hydroxyl could interact with the C-terminal Phe8 carboxylate either directly or via a relay mechanism provided by a His4 imidazole bridge. These two conformers are also recreated when AngII binds to AT_1_R, and may reflect inverse agonist and agonist binding modes, respectively [65,66].

The discovery of ARB sartans launched a new era in angiotensin research, aided by crystal structures for the AT_1_R bound to peptide as well as nonpeptide ligands. Principal interactions involve receptor-based Lys199 for AngII peptides, whereas nearby Arg167 appears to anchor ARBs, suggesting that sartans bind to an overlapping binding site which probably compromises binding of AngII peptides while engendering an inverse agonist response mechanism; however, it differs from that occupied by AngII peptides like sarilesin. Taken together, these findings illustrate the complexity of highly flexible peptides versus rigid nonpeptides for receptor interactions and the permissive nature of mechanisms for inverse agonism at GPCR. Nonpeptide sartans, which tend to be inverse agonists, may also lack the length and flexibility required to promote cooperatively between AT_1_R dimers with G-protein in order to activate the contractile response [27,28,29,30].

Similar findings are observed with opioid receptors, where peptides enkephalin and endorphins exhibit agonist and inverse agonist activities with affinities several orders of magnitude higher than for nonpeptide ligands (morphine, fentanyl). As with AT_1_R, nonpeptide opioid ligands likely interact with a different receptor site from opioid peptides to evoke inverse agonism. Notably, morphine and fentanyl have similar affinities but have markedly different potencies, suggesting that nonpeptide opiates can have substantially different efficacies, along with corresponding dependencies. A comprehensive understanding of these receptor mechanisms is crucial for addressing dependency on addictive drugs [15].

### 3.9. Pharmacokinetic Bottlenecks, Central Exposure, and Safety Considerations

While multi-target GPCR engagement by sartans presents an intriguing repurposing hypothesis, clinical translation is fundamentally governed by pharmacokinetic (PK) feasibility, receptor occupancy, and safety margins. Marketed ARBs are hydrophilic or moderately lipophilic acids that are highly bound to plasma proteins (>98%) and primarily engineered for systemic vascular retention. Consequently, their baseline blood–brain barrier (BBB) penetrance is low under non-inflamed conditions. Proposed applications in substance use disorders and neuro-inflammation would necessitate sustained central nervous system (CNS) exposure. For bisartans such as ACC519TT, the incorporation of dual bulky biphenyl tetrazole moieties significantly increases molecular weight and topological polar surface area, factors that conventionally disfavor passive transcellular permeability across the BBB.

Furthermore, off-target promiscuity at α-adrenergic and opioid receptors must be weighed against cardiovascular homeostasis. Dosing conventional sartans or novel bisartans at levels required to achieve adequate occupancy of lower-affinity off-targets (μOR, δOR) risks provoking profound systemic hypotension, renal hypoperfusion, and hyperkalemia via high-affinity AT_1_R blockade. In vulnerable populations, established ARB toxicological liabilities—including strict teratogenic contraindications during pregnancy and risks in acute renal compromise—remain critical boundaries. For newly developed bisartans, systematic ADMET profiling, metabolic clearance evaluation, and in vivo toxicological screening remain completely uncharacterized and are prerequisites prior to asserting translational viability.

### 3.10. Limitations

Despite the significant insights afforded by computational modeling and network-based pharmacology, several methodological and interpretive limitations must be acknowledged.

#### 3.10.1. Static Receptor Models in Docking Studies

CAD studies typically employ static receptor conformations [96,97], representing uncoupled or inactive receptor states. These models do not account for the dynamic conformational transitions associated with receptor activation, such as shifts from R_inactive_ to R_active_ conformations [98], nor do they incorporate intracellular effectors, such as G-proteins or β-arrestins. Consequently, predicted binding affinities may not fully reflect the ligand’s pharmacodynamics in a physiologically coupled system.

#### 3.10.2. Timescale Constrains in MD Simulations

Although MD simulations provide valuable information regarding ligand–receptor stability and local structural rearrangements, they are generally limited to nanosecond-to-microsecond timescales (e.g., 150–200 ns) [99,100]. However, key conformational events involved in receptor activation, desensitization, or internalization may occur over longer biological timescales [101], potentially limiting the interpretability of simulation data.

#### 3.10.3. Approximations in Binding Energy Calculations

The predictive power of docking simulations is inherently constrained by the approximations of scoring functions and energy models [102]. Many of these algorithms incompletely account for entropic contributions to binding free energy [103], leading to potential discrepancies between computed scores and experimentally observed affinities. Accordingly, docking results should be interpreted as semi-quantitative rather than absolute indicators of binding strength.

#### 3.10.4. Binding Affinity Does Not Imply Functional Activity

High predicted affinity in silico does not necessarily translate to biological efficacy in vitro or in vivo [104]. Some high-affinity interactions may be pharmacologically silent, while low-affinity binding, especially at allosteric or noncanonical sites, may elicit meaningful functional effects. Thus, experimental validation through functional assays remains essential to confirm predicted bioactivity [105].

#### 3.10.5. Complexity of Network-Based Predictions

Network pharmacology frameworks draw upon large-scale omics data to infer drug–target–disease associations [106,107]. However, these models often lack critical biological features such as tissue-specific expression, post-translational modifications, signaling feedback, and compensatory mechanisms [108]. As such, predictions may not fully recapitulate biological complexity, particularly under disease-specific or homeostatic conditions.

#### 3.10.6. Absence of Individualized Clinical Context

Most computational analyses do not incorporate inter-individual variability arising from genetic polymorphisms, comorbidities, or pharmacokinetic differences. These patient-specific factors can profoundly influence both drug response and safety, thereby limiting the generalizability of in silico predictions to diverse clinical populations.

## 4. Conclusions

This review discusses the in silico and systems-level interactome data indicating putative crossover binding profiles for ARBs and structurally modified bisartans beyond their primary target. Beyond their canonical interaction with the AT_1_R, these compounds demonstrate high predicted affinities for αARs and μORs, positioning them as pharmacologically versatile ligands capable of modulating multiple GPCR families.

The observed receptor promiscuity arises from the shared structural features of ARBs, particularly their anionic tetrazole moieties, cationic aromatic cores, and hydrophobic scaffolds, which enable diverse interaction modes across orthosteric and potentially allosteric binding sites. Molecular dynamics simulations further support the stability of these interactions and reveal receptor-specific conformational adaptations that may underlie differential signaling outcomes.

In the broader context of proteomics and drug repurposing, these findings underscore the importance of integrating ligand–receptor structural modeling with interactome-level analysis. The ability of ARBs to engage noncanonical targets involved in immune modulation, neurotransmission, and viral entry highlights their potential utility in treating conditions, such as addiction, neuro-inflammatory diseases, and viral infections (e.g., COVID-19). Moreover, their selective engagement with desensitized receptor states suggests a promising mechanistic avenue for developing non-addictive modulators of GPCR signaling.

Nevertheless, the limitations of static receptor models, short molecular dynamics timescales, and qualitative binding predictions reinforce the need for experimental validation, particularly through functional proteomics and pharmacodynamic studies. Future work should aim to profile ARB interactions with receptor proteoforms and post-translationally modified states using high-resolution structural and mass spectrometry-based approaches.

In conclusion, sartans and bisartans represent versatile chemical scaffolds with theoretical candidacy for therapeutic repurposing, subject to downstream experimental and clinical verification. Their ability to bind multiple targets, along with favorable pharmacokinetics and clinical familiarity, supports further research in precision medicine and systems pharmacology frameworks. Unlocking their full clinical potential will require integrating computational predictions with experimental proteomic validation.

## Figures and Tables

**Table 1:** Comparative profiling of individual sartans and bisartans: Targets, level of supporting evidence, functional outcomes, and translational bottlenecks. Chemical structures were created in BioRender. Gadanec, L. (2026) https://BioRender.com/4vs635g (accessed on 25 September 2026).

Compound	Chemical Structure	Scaffold/Structural Characteristics	Proposed Targets	Evidence Level	Biological/Functional Outcome	Translational Feasibility and Limitations
**ACC519TT**	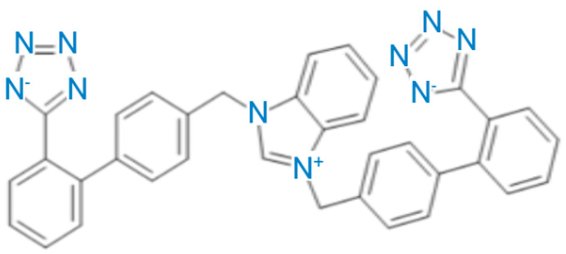	Benzimidazole core; symmetric di-biphenyl tetrazole	AT_1_R; α1AR; μOR; δOR; and viral arginine clusters [15,45]	Exclusively in silico (targeted docking and 150–200 ns MD) [15]	Putative multi-target GPCR engagement via electrostatic and π-π contacts [15,45]	High MW (>600); unknown BBB permeability; unverified ADMET and lack of functional bioassays [15]
**Candesartan**	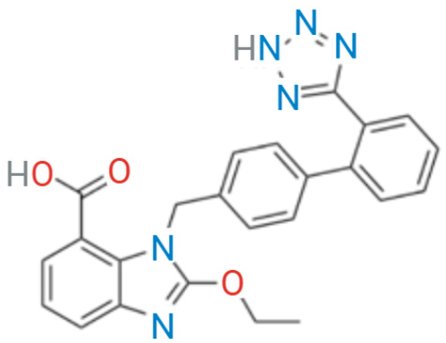	Benzimidazole core; ethoxy substituent; single tetrazole	AT_1_R; PPARγ; Microglial neuro-inflammation [37]	In vitro and in vivo (rodent microglia; 1–5 µM) [37]	Suppresses TNF-α, IL-6, IL-18 via PPARγ/AMPK pathway [37]	Requires high local concentrations; minimal CNS bioavailability without optimized delivery systems [37]
**Irbesartan**	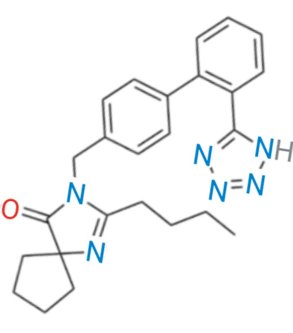	Spiro-cyclopentyl imidazolinone; single tetrazole	AT1R; c-JUN/Hippo/YAP1 axis [41]	In vitro and preclinical (oncology models) [41]	Reverses gemcitabine resistance; downregulates iron metabolism [41]	Oncological utility remains experimental; distinct from primary hemodynamic mechanism [41]
**Losartan**	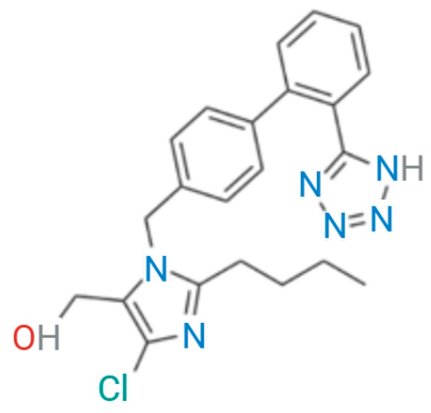	Imidazole core; monochlorinated; single biphenyl tetrazole	AT_1_R (canonical); SARS-CoV-2 Mpro/ACE2 [13,38]	In vitro/clinical (cardiovascular); in silico/in vitro (viral) [13]	Lowers BP; modest viral protease inhibition predicted [13]	Low BBB penetration; clinical COVID-19 trials showed conflicting therapeutic benefits [46]
**Telmisartan**	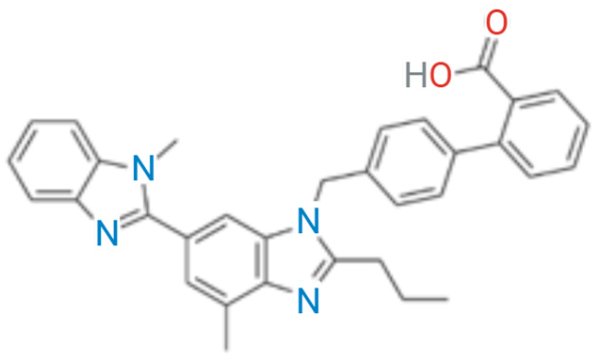	Bis-benzimidazole core; lipophilic carboxylate (no tetrazole)	AT_1_R; PPARγ (partial agonist); glioma AT1R [42,47]	In vitro and in vivo (µM EC_50_ for PPARγ) [42]	Modulates metabolic target genes; inhibits glioma growth [42,47]	High lipophilicity aids tissue uptake; off-target GPCR binding remains largely unverified in vitro [42]
**Valsartan**	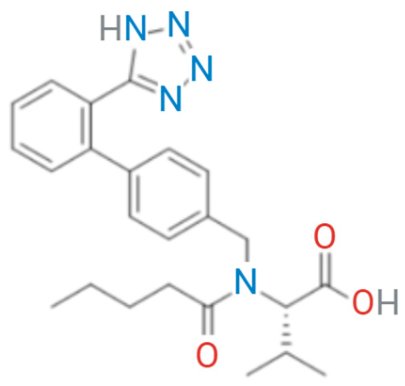	Non-heterocyclic acylated amino acid; single tetrazole	AT_1_R; morphine withdrawal pathways [36]	In vivo (rodent co-treatment; 20 mg/kg) [36]	Attenuates signs of morphine tolerance and physical withdrawal jumps [36]	Mechanism likely mediated via downstream systemic/central RAAS blockade rather than direct opioid [36]

## Data Availability

No new data were created or analyzed in this study. Data sharing is not applicable to this article.

## Abbreviations

The following abbreviations are used in this manuscript:

ABI3	ABI family member
ACC519TT	Benzimidazole bis-N,N′-biphenyl tetrazole
ACE	Angiotensin-converting enzyme
AGTRAP	Angiotensin II receptor-associated protein
ALG10	Alpha-1,2-glucosyltransferase ALG10-A
AngII	Angiotensin II
AP2M1	Adaptor-related protein complex 2 subunit Mu 1
AR	Adrenergic receptor
ARB	Angiotensin receptor blocker
ARFGAP1	Adenosine diphosphate-ribosylation factor guanosine triphosphatase-activating protein 1
ARHGDIG	Rho guanosine nucleotide-dissociation inhibitor 3
ARRB1	Beta-arrestin 1
ARRB2	Beta-arrestin 2
ATP1B1	Sodium/potassium-transporting adenosine triphosphatase subunit beta-1
ATP2B3	Plasma membrane calcium-transporting adenosine triphosphatase 3
ATP2B4	Plasma membrane calcium-transporting adenosine triphosphatase 4
ATP12A	Potassium-transporting adenosine triphosphatase alpha chain 2
ATP13A1	Endoplasmic reticulum transmembrane helix translocase
AT_1_R	Angiotensin II type 1 receptor
BAALC	Brain and acute leukemia cytoplasmic protein
BASP1	Brain acid soluble protein 1
BBB	Blood–brain barrier
BDKRB2	B2 bradykinin receptor
BNIP3	B-cell lymphoma 2/adenovirus E1B 19 kDa protein-interacting protein 3
BRD8	Bromodomain-containing protein 8
CAD	Computer-aided drug docking
CAV1	Caveolin-1
CBR1	Carbonyl reductase 1
CCDC25	Coiled-coil domain-containing protein 25
CD37	Leukocyte antigen CD37
CERS6	Ceramide synthase 6
CLCC1	Chloride intracellular channel 1
CNS	Central nervous system
COVID-19	Coronavirus disease 2019
CRS	Charge relay system
CUEDC2	CUE domain-containing protein 2
c-JUN	Jun proto-oncogene, AP-1 transcription factor subunit
C1ORF112	Chromosome 1 open reading frame 112
DBNDD2	Dysbindin domain-containing protein 2
DCX	Doublecortin
DNAJC8	DnaJ heat shock protein family (Hsp40) member C8
DDX3Y	Adenosine triphosphate-dependent ribonucleic acid helicase
DYRK1A	Dual specificity tyrosine-phosphorylation-regulated kinase 1A
ENDOD1	Endonuclease domain-containing 1 protein
FAM13B	Protein FAM13B
FBXW11	F-box/WD repeat-containing protein 11
FNBP1	Formin-binding protein 1
GHITM	Growth hormone-inducible transmembrane protein
GNA12	Guanine nucleotide-binding protein subunit alpha-12
GNB2L1	Receptor for activated C kinase 1
GOT1	Glutamic-oxaloacetic transaminase 1
GPAA1	Glycosylphosphatidylinositol anchor attachment 1 protein
GPCR	G-protein-coupled receptor
GRK5	G-protein-coupled receptor kinase 5
HDGFRP2	Hepatoma-derived growth factor-related protein 2
HHATL	Protein-cysteine N-palmitoyltransferase HHAT-like protein
HTR2B	5-hydroxytryptamine receptor 2B
JAK1	Janus kinase 1
JAK2	Janus kinase 2
LLGL1	JKAMP, mitogen-activated protein kinase 8-associated membrane protein
LCNL1	Lipocalin-like 1 protein
LLGL	Scribble cell polarity complex component 1
LZTS1	Leucine zipper tumor suppressor 1
MAPK1	Mitogen-activated protein kinase 1
MD	Molecular dynamics
NACAD	NAC-alpha domain-containing protein 1
NDRG2	N-myc downstream-regulated gene 2
NPTN	Neuroplastin
NUP88	Nucleoporin 88
OAZ1	Ornithine decarboxylase antizyme 1
OR	Opioid receptors
PCMT1	Protein-L-isoaspartate(D-aspartate) O-methyltransferase
PDE6H	Retinal cone rhodopsin-sensitive cyclic guanosine monophosphate 3′,5′-cyclic phosphodiesterase subunit gamma
PIGU	Glycosylphosphatidylinositol-anchor transamidase component
PLCH2	1-phosphatidylinositol 4,5-bisphosphate phosphodiesterase beta-2
PNMA1	Paraneoplastic antigen Ma1
PPARγ	Peroxisome proliferator-activated receptor gamma
PPP1R3E	Protein phosphatase 1 regulatory subunit 3F
PSMD10	26S proteasome non-adenosine triphosphatase regulatory subunit 10
PTGFR	Prostaglandin F2-alpha receptor
PTGS2	Prostaglandin-endoperoxide synthase 2
RAB4A	Ras-related protein Rab-4A
RAB5A	Ras-related protein Rab-5A
RAB7A	Ras-related protein Rab-7a
RAB11A	Ras-related protein Rab-11A
R_active_	Active signaling state
RAF1	Raf-1 proto-oncogene, serine/threonine kinase
RAAS	Renin–angiotensin–aldosterone system
RBSN	Rabenosyn-5RAB4A
R_des_	Desensitized/internalized state
R_inactive_	Basal/inactive ground state
RMSD	Root-mean-square deviation
RTN3	Reticulon-3
SAE1	SUMO-activating enzyme subunit 1
SARS-CoV-2	Severe acute respiratory syndrome coronavirus 2
SCG5	Neuroendocrine protein 7B2
SEP15	Selenoprotein F
SERINC3	Serine incorporator 3
SETD7	Histone-lysine N-methyltransferase
SPG21	Maspardin
SLC6A8	Sodium- and chloride-dependent creatine transporter 1
SLC16A1	Monocarboxylate transporter 1
SLC19A2	Thiamine transporter 1
THOC7	THO Complex Subunit 7
TMEM11	Transmembrane protein 11
TMEM161A	Transmembrane protein 161A
TSPAN15	Tetraspanin 15
TMEM80	Transmembrane protein 80
UBR4	E3 ubiquitin-protein ligase component n-recognin 4
UGT8	2-hydroxyacylsphingosine 1-beta-galactosyltransferase
VAC14	Protein VAC14 homolog
VKORC1L1	Vitamin K epoxide reductase complex subunit 1-like 1
YWHAQ	14-3-3 protein theta
Å	Angstrom
μOR	mu-opioid receptor
δOR	delta-opioid receptor
